# Investigating the attenuated psychosis syndrome in youth with autism spectrum disorder: results from an observational study

**DOI:** 10.3389/fpsyt.2025.1536361

**Published:** 2025-03-31

**Authors:** Assia Riccioni, Maria Pontillo, Lenardo Emberti Gialloreti, Mariagrazia Cicala, Michelangelo Vasta, Mattia Gatto, Lucrezia Arturi, Martina Siracusano, Michelangelo Di Luzio, Stefano Vicari, Luigi Mazzone

**Affiliations:** ^1^ Child Neurology and Psychiatry Unit, Tor Vergata University Hospital, Rome, Italy; ^2^ Child and Adolescent Neuropsychiatry Unit, Bambino Gesù Children’s Hospital, IRCCS, Rome, Italy; ^3^ Department of Biomedicine and Prevention, University of Rome Tor Vergata, Rome, Italy; ^4^ Department of Systems Medicine, University of Rome Tor Vergata, Rome, Italy; ^5^ Department of Education, Cultural Heritage and Tourism, University of Macerata, Macerata, Italy; ^6^ Department of Life Sciences and Public Health, Università Cattolica Del Sacro Cuore, Rome, Italy

**Keywords:** clinical high risk, prodrome, psychosis, neurodevelopment, schizophrenia, autism

## Abstract

**Introduction:**

Despite evidence suggesting increased rates of psychosis in individuals with autism spectrum disorder (ASD), the detection of prodromal psychotic symptoms, including attenuated psychosis syndrome (APS), remains underexplored in this population.

**Methods:**

The primary aim of the present study was to characterize the clinical phenotype of young individuals with ASD who also present with APS (ASD/APS; n = 48) in comparison with individuals with APS only (n = 93) and those with ASD only (n = 30) (age range 9–23 years). Assessments included standardized measures of autistic symptoms (Autism Diagnostic Observation Schedule–Second Edition; ADOS-2), pre-psychotic symptoms (Structured Interview for Psychosis-Risk Syndromes; SIPS), and cognitive and adaptive functioning.

**Results:**

Overall, the ASD/APS group demonstrated significantly poorer general adaptive skills compared with the APS group (p = 0.006) and the ASD group (p = 0.005). Compared with the APS group, the ASD/APS group exhibited lower scores across all SIPS domains, with the exception of SIPS-P1 (unusual thought content/delusional ideas; p = 0.062; t = −1.882; F = 5.44) and SIPS-P3 (grandiosity; p = 0.156; t = −1.435; F = 22.6). In contrast, the ASD/APS group displayed significantly higher scores in the repetitive and restricted behavior domain compared with the ASD group (p < 0.001). Notably, there were no significant differences in the age of APS onset across groups (p = 0.601; t = 0.525; F = 0.253).

**Discussion:**

These findings provide a more nuanced characterization of APS features in individuals with ASD and emphasize the importance of screening for APS in this population, particularly those considered at increased risk. Early detection and intervention could facilitate timely therapeutic support, potentially improving long-term outcomes for these individuals.

## Introduction

Adolescence (10–19 years) (1) is a critical developmental period for the emergence of numerous premorbid “red flags” associated with severe mental illnesses, including psychosis (2). Psychotic disorders are often preceded by atypical developmental trajectories (3, 4) and a prodromal phase referred to as Clinical High-Risk for Psychosis (CHR-P) (5). CHR-P describes a clinical condition characterized by subthreshold psychotic symptoms, associated with an increased risk of developing a full psychotic disorder within the subsequent 2–5 years (6–8).

The CHR-P framework encompasses three distinct clinical subgroups: Attenuated Psychotic Syndrome (APS), Brief (and Limited) Intermittent Psychotic Symptoms (BLIPS or BIPS), and Genetic Risk and Deterioration Syndrome (GRD) (9, 10). Among these, APS is the most prevalent (11) and is currently recognized as the strongest clinical predictor of conversion to psychosis in the general population (12). Introduced in the *Diagnostic and Statistical Manual of Mental Disorders-Fifth Edition* (DSM-5) Research Appendix (Section III) in 2013, APS is characterized by attenuated delusions, hallucinations, or disorganized speech that occur at least once per week over the past month but are not severe enough to meet the diagnostic threshold for a psychotic disorder (13).

Detection of APS plays a pivotal role in identifying CHR-P status and enabling timely preventive interventions, which can reduce the likelihood of progression to full-blown psychosis and improve long-term outcomes (5, 6). Early identification of APS is particularly important for vulnerable populations with preexisting conditions associated with an elevated risk of severe mental illness, such as autism spectrum disorder (ASD) (14–16).

ASD is a lifelong neurodevelopmental condition characterized by impairments in social-communicative skills and the presence of restricted and stereotyped patterns of behavior and interests (13). Individuals with ASD are known to be at an increased risk for developing one or more psychiatric disorders over their lifetime (17–19). Among these psychiatric co-occurring conditions, psychotic disorders are reported at varying rates, ranging from 0.6% (95% CI = 0.3–1.1) (20–22) to 9.4% (95% CI = 7.52–11.72) in adult populations (23). Moreover, research has shown that individuals with ASD are not only at increased risk for concomitant psychotic symptoms, but that individuals with psychotic disorders may also exhibit higher levels of autistic traits (24).

Thus, despite ASD and psychosis being distinct conditions, there is an emerging hypothesis suggesting a continuum between them (25, 26). This hypothesis is supported by evidence of shared neurodevelopmental origins and overlapping clinical features, particularly related to impairments in social and communicative skills (21, 27–29). Notably, core ASD symptoms—such as reduced eye contact, poor emotional and social reciprocity, and stereotyped language—can resemble psychotic symptoms, including social withdrawal, blunted affect, and disorganized speech (16, 27, 29). Consequently, identifying and characterizing psychotic symptoms in individuals with ASD remains challenging (4, 16, 27), further complicated by evidence that the presentation of psychotic symptoms in ASD may differ from that observed in the general population, often exhibiting a greater emphasis on affective symptoms, a more acute onset, and a transient course (15). As a result, the detection and characterization of psychotic symptoms—especially during the prodromal phase—remains a significant challenge in individuals with ASD (16, 29). Therefore, while available studies indicate an increased prevalence of attenuated psychotic symptoms (APS) in ASD (30–34), it remains unclear whether prodromal symptoms in ASD mirror those experienced by the general population (16, 30, 33, 35, 36). In response, research on the overlap and semiology of prodromal psychotic symptoms in ASD is rapidly expanding (26, 28, 30, 33–35).

In this context, we recently conducted a preliminary study (33) to examine autistic and psychotic symptoms, as well as cognitive and adaptive skill profiles, in a sample of young individuals with ASD (ages 10–23 years) with (n = 13) or without (n = 18) concomitant APS. Our findings revealed that individuals with ASD/APS exhibited more severe autistic symptoms, greater social skills impairments (in social awareness and social cognition), and worse general adaptive functioning compared with the ASD-only group. Furthermore, the primary differences in psychotic symptoms between the ASD/APS and ASD groups were observed in the domains of positive and disorganized symptoms, supporting the notion that the overlap between ASD and psychosis is more pronounced in negative symptoms rather than positive ones (4, 16, 37). However, the relatively small sample size (n = 31) and the lack of an APS-only control group limited our ability to fully explore and characterize the differences in social skills impairment and psychotic symptom profiles between individuals with ASD and those in the general population. Nevertheless, disseminating knowledge about the clinical characteristics of APS in ASD is crucial for informing clinical prognosis and therapeutic strategies for individuals with ASD.

It is worth noting that, to date, there is a paucity of observational empirical studies on this topic. Most existing data come from retrospective studies (29, 30, 36) or involve small sample sizes (29, 33), limiting the ability to draw definitive conclusions. Moreover, no previous studies have directly compared the clinical profiles of individuals with ASD/APS to those with APS (without ASD) and ASD alone.

Therefore, the primary aim of the present study was to explore and characterize the clinical profile of a sample of young individuals with ASD presenting concomitant APS, in comparison with individuals with APS alone (without ASD), aged 9–23 years. This was achieved through the administration of standardized, gold-standard assessments of both autistic and psychotic symptoms, as well as cognitive and adaptive functioning.

## Materials and methods

### Procedure

This is an observational cohort study conducted in the context of previous research projects (33). The study was approved by the Independent Ethical Committee of the University Hospital, Fondazione Policlinico Tor Vergata (Register number 126/18), and informed consent was obtained from all legal holders of custody.

#### Participants

Our sample was constituted by individuals (age range 9–23 years) recruited from the Child Psychiatry Unit of the University of Rome Tor Vergata Hospital and from the Child and Adolescent Neuropsychiatry Unit of the Bambino Gesù Children’s Hospital, between January 2019 and September 2023. Specifically, the participants included in the present study were assessed for their eligibility by a multidisciplinary team (child psychiatrists and psychologist). To minimize the risk of symptom misinterpretation or underestimation, the clinical assessments were conducted by expert clinicians with specialized knowledge in both autism and psychosis.

In order to be eligible, participants were required to have (1) a condition of Attenuated Psychosis Syndrome (APS), considered confirmed with a score of 3, 4, of 5 on the Structured Interview for Psychosis-Risk Syndromes (SIPS) (38) and/or (2) a diagnosis of Autism Spectrum Disorder (ASD) without language and/or cognitive impairment (Intelligence Quotient - IQ above 70), performed on the basis of the Diagnostic and Statistical Manual of Mental Disorders–Fifth Edition (DSM–5) or the Diagnostic and Statistical Manual of Mental Disorders, Fifth Edition, Text Revision (DSM-5 TR) criteria (13), supported by the administration of the Autism Diagnostic Observation Schedule–Second Edition (ADOS–2) (39).

The adopted exclusion criteria were the presence of IQ equal or below 70, non-fluent speech, epilepsy, and other concurrent psychiatric or neurodevelopmental conditions (e.g., obsessive–compulsive disorder, attention deficit, and hyperactivity disorder).

A comprehensive clinical assessment of cognitive and adaptive skills, as well as of autistic and psychotic symptoms, was performed as described below.

### Materials

#### Cognitive skills assessment

Based on age and each individual’s ability to cooperate, all individuals included in our sample underwent a non-verbal or verbal cognitive evaluation to assess the IQ. Specifically, the Leiter International Performance Scale-Revised (Leiter-R) (40), which is not reliant on verbal skills, was chosen for children and adolescents with more severe communications impairments and limited levels of cooperation. Otherwise, the Wechsler Intelligence Scale for Children-fourth Edition (WISC-IV) (41) or the Wechsler Adult Intelligence Scale-Revised (WAIS-R) (42) tests, including verbal language in the assessment of IQ, were used.

For all of these scales, raw scores were converted into composite scores, and a mean and standard deviation (SD) IQ value of 100 ± 15 was considered.

#### Adaptive skills assessment

The Adaptive Behavior Assessment System, Second Edition (ABAS-II) (43), was administered to parents of all included individuals. In particular, the “5–21 years” ABAS-II form was used. Parents were asked to rate the child’s skills to complete an activity (from 0 = “not able to” to 3 = “able to do it and always performs it when needed”) in regard to 10 functioning areas (i.e., communication, use of the environment, preschool competences, domestic behavior, health and safety, play, self-care, self-control, social abilities, and motility). The questionnaire provides three main adaptive domains: conceptual (CAD), practical (PAD), social (SAD), and a comprehensive score, General Adaptive Composite (GAC). Each of these indexes is standardized with a mean of 100 and an SD of 15.

#### Psychotic symptoms assessment

The Structured Interview for Psychosis-Risk Syndromes (SIPS) (38) was administrated by expert clinicians to all included individuals. The SIPS is a semi-structured interview, which rates along four major symptom dimensions on the Scale of Prodromal Symptoms (SOPS) among four symptom domains: positive symptoms (SIPS-P items P1–P5: P1 unusual thought content; P2 suspiciousness; P3 grandiosity; P4 perceptual abnormalities; and P5 disorganized communication), negative symptoms (SIPS-N items N1–N6: N1 social anhedonia; N2 avolition; N3 expression of emotion; N4 experience of emotions and self; N5 ideational richness; and N6 occupational functioning), disorganized symptoms (SIPS-D items D1–D4: D1 odd behavior or appearance; D2 bizarre thinking; D3 trouble with focus and attention; and D4 impaired personal hygiene), and general symptoms (SIPS-G items G1–G4: G1 sleep disturbance; G2 dysphoric mood; G3 motor disturbances; and G4 impaired tolerance to normal stress). Each item has a severity scale ranging from 0 (Absent) to 6 (Severe/Extreme).

Based on the SIPS/SOPS criteria, the presence of an APS condition is confirmed with a score of 3, 4, of 5 on the SIPS positive symptoms scale (SIPS-P) (12, 38, 44).

For the purpose of the present study, both the total and the single-item scores for each SIPS subscale (SIPS-P, SIPS-N, SIPS-D, SIPS-G, and SIPS total score) were analyzed.

#### Autistic symptoms assessment

The Autism Diagnostic Observation Schedule–Second Edition - ADOS-2 (39) was administered to all included individuals. The ADOS-2 is a semi-structured observational tool considered as the gold standard for the assessment of autistic symptoms. It includes five modules (Toddler, 1, 2, 3, and 4) selected on the basis of age and expressive language level. The ADOS-2 algorithm is organized in Social Affect (SA), Restricted and Repetitive Behaviors (RRB), and the total score (TOT). Modules 1, 2, and 3 provide the Calibrated Severity Score (CSS), ranging from 1 to 10, which indicates autism symptom severity. Module 4 is used with verbally fluent adults who are likely to demonstrate a wide range of abilities. For the present study, module 3 and module 4 were administered.

Even if module 4 does not provide a CSS, a revised algorithm is available in order to provide a calibrated score that can be compared with algorithms used for ADOS-2 Modules 1–3 (45).

### Statistical analysis

A descriptive analysis was performed to report the number of subjects in each group, the male–female ratio, and the means ± SD of the subjects’ age. Differences in age, gender, IQ, adaptive functioning, autistic symptom levels, and psychotic symptoms between groups were assessed using independent samples t-tests, Mann–Whitney tests, independent samples Kruskal–Wallis tests, one-way ANOVA with *post-hoc* analysis adjusted for multiple comparisons (Tukey HSD), and Pearson chi-squared tests, where appropriate. Bivariate Spearman’s correlations were applied to estimate the relationships between SIPS subscale scores and the IQ values. Results are presented as number of observations and percentages or means ± SD. An alpha level of 0.05 was used for all statistical analyses. All statistical analyses were performed using SPSS v.26.0 (IBM Corp., Armonk, NY, USA).

## Results

A final sample of n=171 individuals was included. Specifically, we included a group of individuals with APS (n= 93; M:F= 47:46; age: 15.3 ± 2.4) in comparison with a group of ASD/APS (n= 48; M:F= 31:17; age: 15.2 ± 2.6). We further included a control group of individuals with ASD (n=30; M:F=25:5; age: 14.8 ± 2.9). Samples’ demographic and clinical data are summarized in **Table 1**.

**Table 1:** Demographic and clinical characteristics of the sample.

Demographic data	ASD (n=30)	ASD/APS (n=48)	APS (n=93)	ASD/APS vs. APS	ASD/APS vs. ASD	APS vs. ASD
	mean ± SD	mean ± SD	mean ± SD	p value	p value	p value
***Age***	14.8 ± 2.9	15.2 ± 2.6	15.3 ± 2.4	0.996	0.759	0.668
***Male/Female***	25/5	31/17	47/46	0.403	0.073	0.501
***IQ***	105.2 ± 18.1	97.8 ± 19.8	102.5 ± 16.5	0.307	0.183	0.754
Mental illness familiarity *Yes/no*	5/25	17/31	36/57	–	–	–
***Age APS onset***	–	13.5 ± 2.0	13.3 ± 2.2	0.601	–	–
Clinical data						
Adaptive skills						
*ABAS_GAC*	77.1 ± 16.6	65.5 ± 15.9	69.2 ± 12.5	**0.006**	**0.005**	0.423
*ABAS_CAD*	85.4 ± 15.5	72.6 ± 15.5	75.3 ± 16.8	0.103	**0.003**	0.060
*ABAS_SAD*	79.8 ± 16.5	66.7 ± 13.1	66.6 ± 14.8	0.107	**<0.001**	**0.008**
*ABAS_PAD*	77.3 ± 18.5	66.2 ± 17.4	72.8 ± 14.6	**0.005**	**0.034**	0.989
Autistic symptoms						
*ADOS-SA*	8.2 ± 2.9	8.3 ± 2.9	0.1 ± 0.4	–	0.591	–
*ADOS_RRB*	1.9 ± 1.8	8.0 ± 2.6	0 ± 0	–	**<0.001**	–
*ADOS_CSS*	6.2 ± 1.5	5.7 ± 2.1	0.1 ± 0.2	**<0.001**	0.234	**<0.001**
Psychotic symptoms						
*SIPS-P*	1.5 ± 1.5	11.9 ± 6.5	16.1± 4.4	**0.002**	–	–
*P1*	–	3.1 ± 0.9	3.4 ± 1.1	0.062	–	–
*P2*	–	2.7 ± 1.3	3.3 ± 0.9	**0.005**	–	–
*P3*	–	1.9 ± 2.1	2.4 ± 1.4	0.156	–	–
*P4*	–	2.0 ± 1.6	3.3 ± 1.4	**<0.001**	–	–
*P5*	–	2.2 ± 1.9	3.7 ± 1.3	**<0.001**	–	–
*SIPS-N*	3.4 ± 2.2	16.5 ± 12.3	25.7 ± 8.7	**<0.001**	–	–
*N1*	–	2.5 ± 1.8	3.8 ± 1.2	**< 0.001**	–	–
*N2*	–	2.4 ± 2.0	3.9 ± 1.5	**< 0.001**	–	–
*N3*	–	3.2 ± 2.3	4.7 ± 1.6	**<0.001**	–	–
*N4*	–	3.1 ± 2.4	4.6 ± 1.8	**< 0.001**	–	–
*N5*	–	2.7 ± 2.3	4.4 ± 1.7	**< 0.001**	–	–
*N6*	–	2.6 ± 1.8	4.3 ± 1.5	**< 0.001**	–	–
*SIPS-D*	1.4 ± 1.3	8.6 ± 5.8	13.3 ± 4.6	**<0.001**	–	–
*D1*	–	2.6 ± 1.8	4.0 ± 1.6	**< 0.001**	–	–
*D2*	–	2.6 ± 1.5	4.0 ± 1.4	**< 0.005**	–	–
*D3*	–	2.1 ± 1.6	2.9 ± 0.8	**0.004**		–
*D4*	–	1.2 ± 1.3	2.4 ± 1.3	**<0.001**	–	–
*SIPS-G*	1.2 ± 1.1	7.7 ± 5.9	11.9 ± 4.2	**<0.001**	–	–
*G1*	–	2.2 ± 1.8	3.6 ± 1.2	**< 0.001**	–	–
*G2*	–	1.6 ± 1.5	2.8 ± 1.5	**< 0.001**	–	–
*G3*	–	1.5 ± 1.3	2.4 ± 1.2	**< 0.001**	–	–
*G4*	–	2.3 ± 1.6	3.2 ± 1.2	**0.001**	–	–

The significance level for the p-value is set at 0.05. Results in bold indicate those that have reached a statistically significant level.

Legend IQ, intelligent quotient; ABAS_GAC, ABAS-II General Adaptive Domain; ABAS_CAD, ABAS-II Conceptual Adaptive Domain; ABAS_SAD, ABAS-II Social Adaptive Domain; ABAS_PAD, ABAS-II Practical Adaptive domain; ADOS-2, Autism Diagnostic Observation Schedule – Second Edition, ADOS-2; ADOS_SA, Social Affect; ADOS_RRB, Restricted and Repetitive Behaviors; ADOS_CSS, Calibrated Severity Score; SIPS, Structured Interview for Psychosis-Risk Syndromes; SIPS-P, positive symptoms domain; P1, unusual thought content; P2, suspiciousness; P3, grandiosity; P4, perceptual abnormalities; P5, disorganized communication; SIPS-N, negative symptoms domain; N1, social anhedonia; N2, avolition; N3, expression of emotion; N4, experience of emotions and self; N5, ideational richness; N6, occupational functioning; SIPS-D, disorganization symptoms domain; D1, odd behavior or appearance; D2, bizarre thinking; D3, trouble with focus and attention; D4, impaired personal hygiene; SIPS-G, general symptoms domain; G1, sleep disturbance; G2, dysphoric mood; G3, motor disturbances; G4, impaired tolerance to normal stress.

No statistically significant differences emerged between groups (APS *vs.* ASD/APS *vs.* ASD) in terms of age (F=0.382, p=0.683), gender (χ^2^ = 3.205, p= 0.073), and IQ (F=1.793, p=0.170).

Nonetheless, the ASD/APS group showed lower scores in the ABAS-II GAC domain, reaching a statistically significant level when compared with both the APS (p= 0.006) and the ASD (p=0.005) groups. By contrast, no statistically significant differences came out in the ABAS-II GAC indexes between the ASD and APS groups (p=0.423), except for the ABAS-II SAD domain, which resulted more impaired in the ASD group (p=0.008).

In terms of psychotic symptoms, the APS group showed higher psychotic symptom level in all SIPS domains when compared with the ASD/APS individuals (SIPS-P: U=2933, p=0.002; SIPS-N: U=3182, p<0.001; SIPS-D: U=3169, p<0.001; SIPS-G: U=3184.5, p<0.001) (for SIPS/SOPS single-item scores, please refer to **Table 1**). Specifically, the APS individuals presented higher scores in all SIPS subitems when compared with the ASD/APS group, except for the SIPS-P P1 (APS *vs.* ASD/APS: 3.4 ± 1.1 vs. 3.1 ± 0.9; U=2671, p=0.03) and SIPS-P P3 items (APS *vs.* ASD/APS: 2.4 ± 1.4 vs. 1.9 ± 2.1; U=2690, p= 0.04). Being in line, the ASD/APS group reached a mean SIPS-P score ≥3 (thus confirming the presence of an APS condition) only in the P1 item (unusual thought content/delusion ideas; 3.1 ± 0.9) (**Table 2**). More in detail, in the ASD/APS group, a score within 3 and 5 (thus confirming an APS condition) was reached in 81.2% in P1, in 60.4% in P2, in 37.5% in P3, in 54.2% in P4, in 50% in P5 (**Table 2**). To note, within the APS and ASD/APS groups, no statistically significant correlation emerged between the SIPS subscales scores and the IQ value (SIPS-P: r=−0.004, p=0.959; SIPS-N: r=−0.114, p= 0.136; SIPS-D: r=0.022, p= 0.779; SIPS-G: r=0.001, p= 0.998) and between the SIPS subscales and the ABAS-II GAC indexes (SIPS-P: r=0.025, p=0.778; SIPS-N: r=0.047, p= 0.598; SIPS-D: r=0.075, p= 0.401; SIPS-G: r=0.148, p= 0.093).

**Table 2:** Frequencies in SIPS-P item scores ≥3 within the ASD/APS and APS groups.

	SIPS-P				
	P1	P2	P3	P4	P5
	*(N)%*	*(N)%*	*(N)%*	*(N)%*	*(N)%*
**ASD/APS**	(39) 81.2	(29) 60.4	(18) 37.5	(26) 54.2	(24) 50
**APS**	(85) 91.3	(86) 92.5	(34) 36.5	(81) 87	(79) 85

Based on the SIPS/SOPS criteria, the presence of APS is confirmed when a score of 3, 4, or 5 on the SIPS positive symptoms scale (SIPS-P) is reached.

SIPS, Structured Interview for Psychosis-Risk Syndromes; SIPS-P, positive symptoms domain; P1, unusual thought content; P2, suspiciousness; P3, grandiosity; P4, perceptual abnormalities; P5, disorganized communication.

**Figure 1:**
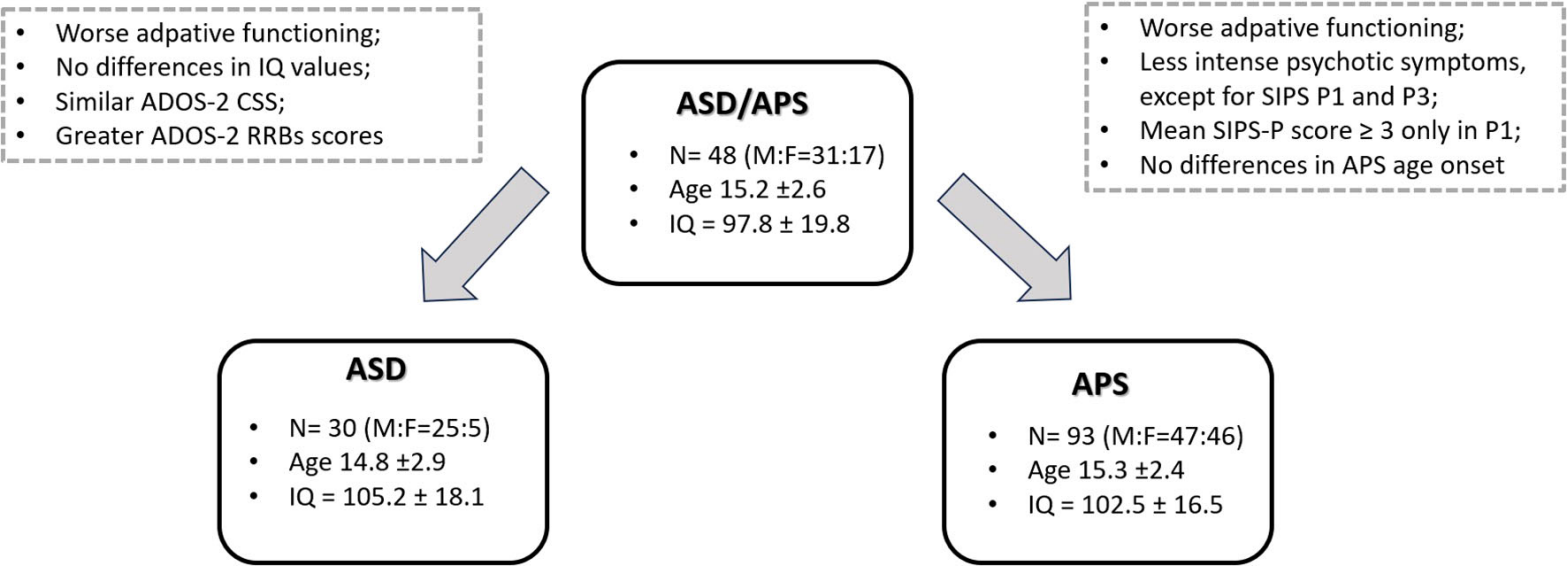
Mean clinical differences between the ASD/APS group and the ASD and APS groups, respectively.

Focusing on the autistic symptoms profile, the mean ADOS 2-CSS score was 6.2 ± 1.5 in the ASD group; 5.7 ± 2.1 in the ASD/APS group and 0.1 ± 0.2 in the APS individuals (**Table 1**). No statistically significant differences emerged in terms of ADOS-2 CSS score (p=0.234) and the ADOS-2_SA domain (p=0.591) between autistic individuals presenting or not presenting concomitant APS (ASD *vs.* ASD/APS). By contrast, the ASD/APS group showed greater scores in the ADOS-2_RRB domain when compared with the ASD (p<0.001) (**Figure 1**).

Within all groups of participants, the *post-hoc* ANOVA model highlighted a negative association between the ADOS-2 CSS and the SIPS-P scores (t=3.006; p=0.004). By contrast, no statistically significant association was found between SIPS-P and IQ (t=1.251; p=0.215), gender (t=0.726; p=0.470), and ABAS-II GAC (t=1.974; p=0.053).

Finally, no statistically significant differences came out in terms of age onset for the APS condition between ASD/APS (13.5 ± 2.0) and the APS individuals (13.3 ± 2.2) (F=0.253; p=0.601).

## Discussion

To the best of our knowledge, this is the first study aimed at characterizing the clinical phenotype of autistic individuals with concomitant APS, in comparison with individuals with APS (without ASD), in terms of autistic and prodromal psychotic symptoms as well as cognitive and adaptive skills, through the administration of gold standard tools. Specifically, we aimed to explore whether individuals with ASD exhibit prodromal psychotic symptoms comparable with those experienced by the general population, with a focus on identifying potential clinical markers for APS in ASD.

Consistent with our previous study (33), our results demonstrated that individuals with ASD/APS exhibited greater impairment in general adaptive functioning skills (ABAS-GAC) compared with those with ASD alone or APS alone. This finding supports the notion that concomitant attenuated psychotic symptoms in autistic individuals may contribute to more significant impairments in adaptive functioning, which could, in turn, negatively affect quality of life and lead to poorer mental health outcomes (19, 46). Notably, data from our previous study (33) also highlighted that individuals with ASD/APS who later converted to full psychosis showed greater baseline impairments in adaptive skills (ABAS-II GAC) compared with those who did not convert. Thus, enhancing adaptive functioning skills in ASD individuals considered at increased risk for APS may play a crucial role in therapeutic strategies and long-term outcomes.

Focusing on the psychotic symptoms assessment, when compared with APS individuals the ASD/APS group overall presented lower scores in all the SIPS domains, except for the SIPS-P1 (*unusual thought content/delusional ideas*) and the SIPS-P3 (*grandiosity*) items. More in detail, in ASD/APS individuals, a mean SIPS-P score ≥3 (thus supporting the APS condition definition) was found only in the SIPS-P1 item. To note, unusual thought content, delusional ideas, and grandiosity are symptoms of disorganized thinking, which refers to disjointed and incoherent thought processes (13). The presence of delusional beliefs and ideation in individuals with ASD has been extensively documented, commonly characterized by “delusions of reference,” “delusions of thought insertion and withdrawal,” and “unusual ideas” (16, 47). Nonetheless, it is important to note that in ASD, individuals could be particularly challenging to distinguish between psychotic features—such as delusional beliefs and disorganized speech—and ASD core symptoms particularly referred to as RRBs, which include stereotyped languages or restricted interests (27). Along this, based on the autistic symptoms profile characterization, ASD/APS individuals included in our sample showed greater scores in the terms of RRBs in comparison with ASD. In this context, it is important to underline that in the present study the clinical assessment was performed by expert clinicians in both autism and psychosis in order to avoid possible symptoms’ under/mis-interpretation. Therefore, we could hypothesize that ASD individuals who presented greater symptoms severity in terms of RRBs could be the one at increased risk for APS, specifically detected by a higher score in the SIPS-P 1 item. As an element of proof that RRBs could play a crucial role, Jutla et al. (3) recently investigated whether previous neurodevelopmental symptoms could predict that an individual at clinical high risk (CHR) will convert to psychosis in a sample of n=151 CHR cohort, showing that Restricted and Repetitive Behaviors during childhood increase the risk of later conversion (OR 13.29, 95% CI 1.5–309.84). Consistent with a transdiagnostic perspective, available studies in the field have highlighted a neurodevelopmental-phenomenological bottom-up model, suggesting that a history of neurodevelopmental RRBs, particularly those related to sensory phenomena, may precede the emergence of obsessive-compulsive behaviors (48, 49), well recognized as a risk factor for psychosis (50–52).

Additionally, across all groups in our study, we found a negative association between the ADOS-2 CSS and SIPS-P scores. This suggests that individuals with more severe autistic symptoms exhibited less pronounced positive psychotic symptoms (independent of IQ, adaptive skills, and gender). This finding supports the hypothesis that positive psychotic symptoms, particularly those related to the SIPS-P1 item, may effectively define the APS condition, even in individuals with ASD.

Finally, it is important to note that no differences in terms of APS age onset came out between ASD/APS and the APS individuals. Despite this, while the APS population generally refers independently to clinicians due to symptoms distress arisen from a previous relatively well-being status, the perception of APS symptoms onset in ASD individuals could be cloudier. Indeed, the attention given to the autistic condition *di per se* could divert focus from possible concomitant conditions, particularly referred to as premorbid psychotic symptoms. As a consequence, clinicians and researchers are strongly invited not only to investigate and screen for APS in ASD individuals considered at increased risk (i.e., poorer adaptive skills, increased RRBs) but also to further explore clinical features of APS in ASD.

Despite our study highlighting several promising findings with possible important implications for daily clinical practice and future research perspectives, our data need to be supported by evidence coming from other studies specifically aimed to define the APS in ASD. Identifying potential clinical markers of APS in ASD could assist clinicians in developing timely and tailored therapeutic and educational interventions for this population.

## Data Availability

The data that support the finding of this study are available on request from the corresponding author AR.
